# Cancer metastasis networks and the prediction of progression patterns

**DOI:** 10.1038/sj.bjc.6605214

**Published:** 2009-08-25

**Authors:** L L Chen, N Blumm, N A Christakis, A-L Barabási, T S Deisboeck

**Affiliations:** 1 Complex Biosystems Modeling Laboratory, Harvard-MIT (HST) Athinoula A Martinos Center for Biomedical Imaging, Massachusetts General Hospital, Charlestown, MA 02129, USA; 2 Department of Physics, Center for Network Science, Northeastern University, Boston, MA 02115, USA; 3 Department of Health Care Policy, Harvard Medical School, Boston, MA 02115, USA; 4 Center for Cancer Systems Biology, Dana–Farber Cancer Institute, Boston, MA 02115, USA

**Keywords:** progression, metastasis, comorbidity networks, prediction

## Abstract

**Background:**

Metastasis patterns in cancer vary both spatially and temporally. Network modelling may allow the incorporation of the temporal dimension in the analysis of these patterns.

**Methods:**

We used Medicare claims of 2 265 167 elderly patients aged ⩾65 years to study the large-scale clinical pattern of metastases. We introduce the concept of a cancer metastasis network, in which nodes represent the primary cancer site and the sites of subsequent metastases, connected by links that measure the strength of co-occurrence.

**Results:**

These cancer metastasis networks capture both temporal and subtle relational information, the dynamics of which differ between cancer types. Using these networks as entities on which the metastatic disease of individual patients may evolve, we show that they may be used, for certain cancer types, to make retrograde predictions of a primary cancer type given a sequence of metastases, as well as anterograde predictions of future sites of metastasis.

**Conclusion:**

Improvements over traditional techniques show that such a network-based modelling approach may be suitable for studying metastasis patterns.

No disease exists in isolation (Goh *et al*, 2007). Whether it is in a predisposing factor or through a shared environment, or whether it is in regards to aetiology or progression, commonality may be shared among diseases within a single individual or within parts of the abstract space of diseases across a population (Barabasi, 2007; Loscalzo *et al*, 2007). Cancer is increasingly recognised not as a single all-encompassing disease, but rather as a multitude of diseases with, in certain cases, surprisingly disparate characteristics (Fearon, 1997; Golub *et al*, 1999). Although this is ostensibly true on a genetic level, the overarching biological and physical mechanisms by which cancer operates nonetheless remain quite similar – one of its hallmarks being the acquisition of the ability to spread to other parts of the body (Hanahan and Weinberg, 2000). Indeed, such metastases are the cause of a majority of cancer-related deaths (Sporn, 1996; Hanahan and Weinberg, 2000; Chambers *et al*, 2002; Gupta and Massagué, 2006).

Although metastasis is important for systemic tumour expansion, it is a highly inefficient process, with millions of cells being required to disseminate to allow for the selection of cells aggressive enough to survive the metastatic cascade (Chambers *et al*, 2002; Gupta and Massagué, 2006). This cascade is a series of sequential steps, which include the shedding of cells directly into the circulatory system or indirectly through the lymphatic system, survival within the circulation followed by extravasation into the new surrounding tissue to initiate growth at a secondary site, and finally induction of angiogenesis to maintain that growth. Only when cells have overcome all these selective barriers do they manifest themselves as clinically visible metastases, or so-called macrometastases (Holmgren *et al*, 1995; Chambers *et al*, 2002; Gupta and Massagué, 2006).

Paget (Paget, 1889) proposed over a century ago that disseminated cancer cells only colonise choice organ microenvironments that are compatible with their growth. This ‘seed and soil’ hypothesis has endured up to this day, largely confirmed through both clinical and laboratory observations. Not only must the ‘soil’, the target organ, harbour a viable niche that can permit, if not facilitate, the initial survival of extravasated cancer cells, but the ‘seeds’ – these extravasated cancer cells – must also have developed the molecular capabilities to effectively colonise the soil (Fidler, 2003; Gupta and Massagué, 2006).

However, blood flow characteristics and the structure of the vascular system may also be an important contributor to metastatic dissemination patterns (Chambers *et al*, 2002; Fidler, 2003). Nonetheless, it was observed through autopsy studies that in breast and prostate cancer, larger numbers of bone metastases than would be expected based on blood flow arguments alone were found (Weiss, 1992). In contrast, fewer numbers of skin metastases than expected were found for bone, stomach, and testicular cancers. It thus appears that some tumour type–organ pairs may be positively disposed toward metastasis formation, some negatively, and some just what blood flow patterns would dictate (Hart, 1982; Zetter, 1990; Weiss, 1992; Fidler, 2003).

To date, study of such organotropic dissemination patterns have relied primarily on autopsies. These have all been relatively small-scale studies (Abrams *et al*, 1950; Weiss *et al*, 1988; Weiss, 1992; Disibio and French, 2008). However, we may now study these patterns using computational methods on large data sets. Disease ‘comorbidity’ can be thought of and analysed as a network (Lee *et al*, 2008). Indeed, cancer contains many manifestations of networks at various levels of organisation, including the genetic (Tavazoie *et al*, 1999; Aggarwal *et al*, 2006), cellular (Vogelstein *et al*, 2000; Irish *et al*, 2004), and phenotypic (Hidalgo *et al*, 2009). We argue that a topographic network for metastases can be constructed as well; here, the nodes represent sites where metastases may arise and links represent the co-occurrence of such metastases. Such a network dynamically evolves as cancer progresses to more advanced stages. One may imagine a sequence of metastatic events in a patient as a trajectory on this dynamic cancer metastasis network. Such a network may yield further insights into the nature and patterns of metastatic dissemination.

Medicare data allow us to look at patterns of metastatic dissemination on a massive scale, across a broad range of cancer types and secondary sites. The ability to do this is aided not only by the sheer size of the data set, but also by the fact that the data are diagnosis driven. As opposed to data derived from autopsy studies, this provides the advantage of being more clinically relevant in terms of patient management – each diagnosed metastasis at a secondary site is recorded as a separate event. In addition, these data give us another dimension, that is, time. However, it is important to note that this data set restricts our patient population to those aged ⩾65 years.

The aim of this study is not to compare cancers by pathological, molecular, or genetic characteristics, as most studies do, but rather to analyse progression dynamics by the anatomical site of origin. By analysing cancer metastasis using networks, we can derive, quantify, and compare the topographical patterns on a large scale. In addition, we can analyse the dynamics of these networks and their structural properties, using them as the basis for the development of better-performing predictive algorithms. Using these networks as entities on which the metastatic disease of individual patients evolve, we hypothesise that we may make retrograde predictions of primary cancer types given a sequence of metastases and anterograde predictions of future sites of metastasis.

## Materials and methods

### Clinical data

We used the so-called Medicare Provider Analysis and Review (MedPAR) records for 1990–1993, containing a comprehensive set of all the Medicare claims of 13 039 018 elderly patients aged ⩾65 years, who were hospitalised during this 4-year period (we excluded the minority of Medicare beneficiaries <65 years). Such records are highly complete and accurate and have been used for epidemiological and other research (Fisher *et al*, 1990; Mitchell *et al*, 1994; Christakis and Allison, 2006); the coverage of the Medicare programme encompasses 35 million beneficiaries (Landon *et al*, 2004). For every hospital visit, up to 10 disease diagnoses are recorded in the International Classification of Diseases version 9 with Clinical Modification (ICD-9-CM) format. We extracted the subset of patients who had at least one diagnosis within the range of 140–239, which represent neoplasms in the ICD-9 classification scheme. This subset contains 2 265 167 patients, with a total of 6 773 633 hospital visits. Of this subset, 1 420 538 patients had only one neoplasm diagnosis, 488 623 had two, and 191 726 had three. The maximum number of neoplasm diagnoses was 17 (two patients). For each patient, we collapsed all neoplasm diagnosis records into a single sequence of diagnoses, along with the number of hospital visits, the number of neoplasm diagnoses, and the follow-up time. Follow-up time was defined here as the length of time from the diagnosis of the primary cancer to the last diagnosis of any disease.

We then separated patients into groups according to the anatomical site of the primary tumour. The ICD-9 scheme codes neoplasms based on anatomical location rather than histology or other pathological characteristics, and thus our grouping is effectively by ICD-9 number. Supplementary Table S1 shows the three-digit ICD-9 codes corresponding to the 43 selected primary cancer types. Certain groups are less specific than others and include more biologically dissimilar tumour types. Certain groups may also contain many more patients than others, reflecting the nonuniform incidence of cancer based on tissue type and anatomical site. Nonetheless, this grouping allows for a reasonably high-resolution categorisation of anatomical sites. For metastasis diagnoses, we used four- and five-digit codes within 196, 197, and 198, which are similarly classified according to anatomical location. Supplementary Table S2 lists the 27 metastatic sites selected, which include lymph nodes, as well as distant tissues and organs.

### Construction of cancer metastasis networks

Patients were censored by overall follow-up time. In other words, at every point of time, only patients with a longer overall follow-up time are considered. This ensures that the analysed patients are still in the system at a particular point of time, and that we can be confident that they have not died. The nodes of a cancer metastasis network represent the distant sites where metastases may arise for a given primary tumour type. The size of each node represents its conditional incidence or hazard. We defined the incidence hazard function as 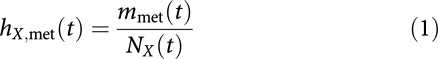 where *m*_met_(*t*) is the number of diagnoses of metastasis met at time *t*, and *N*_*X*_(*t*) is the number of patients remaining at time *t* (where all the patients with an overall follow-up time less than *t* are censored) for primary tumour type *X*. We used discrete times of 1 month, so therefore *t=t*_*i*_*–t*_*i–*1_,*i*=0…48. The cumulative hazard for an *X* and *met* pair is then simply: 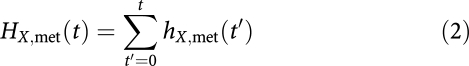 To quantify the dynamics of metastasis development, we looked at the incidence of metastases in terms of co-occurrence at every point of time. This allows us to establish links between the primary tumour and metastasis sites, as well as between different metastasis sites for multiple cases.

### Co-occurrence measures

We quantified co-occurrence using two measures, the *φ*-correlation (Pearson's correlation between dichotomous variables) and relative risk (*RR*). The *φ*-correlation is defined as:


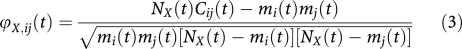
 where *C*_ij_(*t*) is the number of co-occurrences at time *t*. *i* and *j* represent particular sites of metastasis or the primary tumour itself (in other words, one may discover links either between the primary tumour and specific sites of metastasis, or between two different sites of metastasis). *X* represents the primary tumour type. *t=t*_*i*_*–t*_*i–*1_,*i*=0…48. *RR* is defined as: 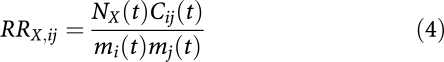 When *i* and *j* are observed together more than random chance would dictate, *RR* >1 and *φ* >0. Although relative risk is used quite commonly in the medical literature, it has certain drawbacks when used in this context. *RR* tends to be biased toward higher values when looking at metastases of low incidence, whereas it is biased toward lower values when looking at those of high incidence. The *φ*-correlation, on the other hand, is biased toward zero when analysing the link between metastases of differing incidence or prevalence. However, *φ* tends to be the better measure for analysing links across multiple cancer metastasis networks, as its scale would fluctuate much less than that of *RR.* This is because the values are better normalised to their respective population sizes, even though the underlying patient population of two primary cancer networks may be quite different.

### Models for predicting the primary cancer site

On the basis of the metastatic patterns, it may be possible to predict the site of a primary, occult cancer. *Multinomial logistic regression*: We first used multinomial logistic regression (MLR) to build an algorithm for predicting the site of a patient's primary cancer, given the vector of their sites of metastases. The data were split into half, with patients randomly assigned to either a training set or a test set. For MLR, we used information only on whether a metastasis at a particular site was detected, disregarding the time at which it was detected. We derived the coefficient estimates with a hierarchical model using the training set, and subsequently applied this model on the test set to assess its accuracy. *Usage of cancer metastasis networks (I)*: We then developed an algorithm using the metastasis networks, incorporating the additional variable of time. Given a sequence of metastases, **M**={*m*_1_(*t*_1_),*m*_2_(*t*_2_),…,*m*_*n*_(*t*_*n*_)}, we define the following matrix:


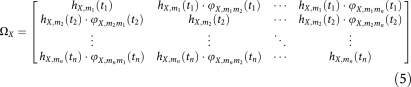
 where *X* denotes the primary cancer type. Each primary cancer site has its own metastasis network, and thus this matrix is a summary of the properties of network *X* at those nodes and links specified by **M**, the temporal sequence of metastases for a single patient. For each patient, the predicted site of their primary cancer is the site *X*, which yields the largest value of ∣∣Ω_*X*_∣∣.

### Models for predicting secondary cancer sites (metastases)

Knowing the primary cancer type, it is clinically important to know how likely it is that metastases may arise and where they may occur, as this will change the staging of the disease, which in turn will guide treatment options. *Fractional method*: In the medical literature, metastasis patterns are often reported as percentages or fractions, without any temporal information. For example, if 30% of patients with breast cancer had or eventually became diagnosed with bone metastases, then for a new breast cancer patient, we will say that this patient has a 30% chance of developing a bone metastasis. For each primary cancer type, we split the patients randomly into either a training set or a test set. Using the training set to derive the fractions of patients developing metastases to each distant site, we then applied those fractions to the test set. We sequentially analysed patients having *n*_mets_=1, 2, 3, and 4 metastases. For each patient with primary cancer type *X* in the test set, the probability of an accurate prediction, *p*_*f*_, is the fraction of patients with primary cancer type *X* in the training set developing *m*_*n*_, given *m*_1_,*m*_2_,…,*m*_*n*–1_. However, we discard this condition by analysing only the *n*th metastasis. That is, using *n*_mets_=3 as an example, we assume *m*_1_ and *m*_2_, so *p*_*f*_ is simply the probability of developing *m*_3_. This allows for more direct comparisons. The overall accuracy is then the mean, *p¯*_*f*_. *Usage of cancer metastasis networks (II)*: With the fractional method as a baseline for comparison, we developed an algorithm for predicting future sites of metastases using cancer metastasis networks. We may think of these networks as entities on which the metastatic disease of individual patients evolve, and are able to incorporate temporal dynamics, as well as subtle relational properties. We developed cancer metastasis networks for each primary cancer type using the training set. For each patient in the test set, the probability of an accurate prediction for *m*_*n*_, *p*_net_, given the primary cancer type *X* and metastases *m*_1_,*m*_2_,…*m*_*n*–1_, is calculated by (see Figure 5A for a graphical summary): 
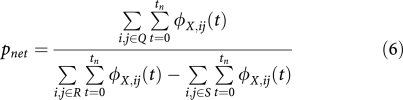
 where *Q* are all the links connecting *m*_*n*_ to the node for the primary cancer site or the nodes *m*_1_,*m*_2_,…*m*_*n*–1_, *R* are all the links from the nodes *m*_1_,*m*_2_,…*m*_*n*–1_ or the primary cancer node, *S* are all the links between any combination of the nodes *m*_1_,*m*_2_,…*m*_*n*–1_ or the primary cancer node, and *t*_*n*_ is the time corresponding to the incidence of metastasis *m*_*n*_. Only *φ*_*X,ij*_(*t*) with *P*-value <0.05 are considered. We analysed separately patients having *n*_mets_=1, 2, 3, and 4 metastases. The overall accuracy is then the mean, *p¯*_net_. The ratio *p¯*_net_/*p¯*_*f*_ captures the improvement over the fractional method of using these networks for prediction.

## Results

### Cancer metastasis networks

We constructed cancer metastasis networks for 43 primary sites, as listed in Supplementary Table S1. We then considered 27 possible secondary sites of dissemination for these primary cancers, as listed in Supplementary Table S2. Nodes represent metastasis sites, and thus number 27 in each network. The incidence of different types of cancer as captured by the data set is shown in Supplementary Figure S1. The largest numbers of diagnoses are for prostate, colon, lung, and bladder cancer. For the majority of cancer primary sites, the pattern of metastatic dissemination sites is quite selective, with a few sites having very strong links and many others holding comparatively weaker links.

### Cancer metastasis network dynamics

**Figure 1:**
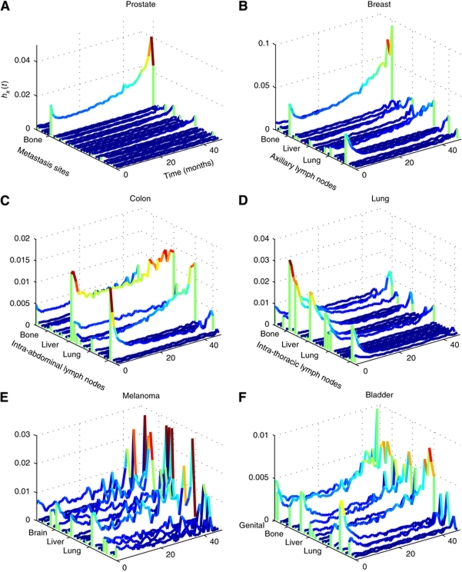
Metastasis incidence hazard functions. Each curve represents an anatomical location at which metastases may arise, showing the dynamics of metastatic progression at that site over time. Displayed are the metastatic progression profiles of six primary tumour types: (**A**), prostate, (**B**), breast, (**C**), colon, (**D**), lung, (**E**), melanoma, and (**F**), bladder. The vector of metastatic sites contains 27 locations, including the lymph nodes, organs, and other anatomical sites. Although labels for the corresponding curves are not shown, this vector of metastatic sites follows the same ordering among these four graphs, thus revealing distinct spatial and dynamical patterns.

**Figure 2:**
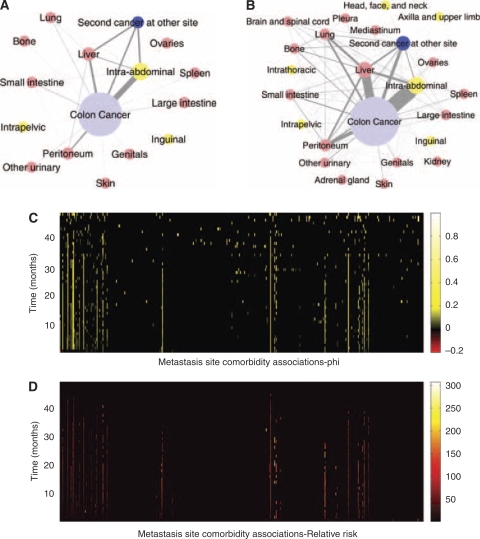
The cancer metastasis network for colon cancer (chosen as a representative example) and the dynamics of its links. (**A**), network at *t*=0, or the time of diagnosis of the primary tumour. (**B**), network at *t*=48 months. Nodes correspond to anatomical sites of metastases, the size of which represents their respective incidence rates. The widths of the links represent the strength of metastasis co-occurrence for two anatomical sites. Yellow nodes represent lymph node metastases; red nodes represent organ metastases. The curves in Figure 1 represent the monthly growth of these nodes, whereas the following (phi) represents the monthly growth of the links: (**C**), metastasis site co-occurrence associations as measured by phi over time. All the possible associations are lined up on the x axis, and their temporal dynamics are represented by the y axis. Only phi with *P*-value <0.01 are shown. (**D**), metastasis site co-occurrence associations as measured by relative risk over time. Only *RR* values with 99% confidence interval or *RR* <0.1 are shown.

Metastasis conditional incidence (hazard) functions for cancers arising at six primary sites are shown in Figure 1. Each curve represents the hazard function for a particular secondary site. Similarly, with the metastasis network links, we can plot their dynamics over time. Figure 2A is the colon cancer-specific metastasis network at *t*=0, and Figure 2B shows the network at *t*=48 months. We can extract dynamical information from the evolution of the network links over time. Figures 2C and D show, for the array of all possible pair-wise links, the monthly increase in the link strength. For any given pair, link strength representing the likelihood metastases at one anatomical site will be found simultaneously with metastases at the other site. Only statistically significant links are shown. Figure 2C, which uses the phi measure to characterise link strength, creates a more detailed picture of the overall dynamics. Initially, a few links steadily and solidly increase. As cancers progress, many more links are added, and link addition becomes much more scattered, and thus covers many more link possibilities. As a consequence, at *t*=0, the strength distribution of these links is narrow and centred about a relatively low strength value (Supplementary Figure S2A). As the cancers progress, these distributions naturally shift toward higher strength values, and evolve toward a more uniform profile.

Using the information on link dynamics for each network, we can then compare the networks and determine how similar they are to one another, across distinct cancer types. This takes into account not only topography but dynamics as well. We measured the pair-wise correlations between metastasis network links for every primary cancer type. The correlation coefficient matrix is shown in Supplementary Figure S3. Although the vast majority of primary cancer types exhibit low correlation values with one another based on this approach, a few do stand out: (i) ‘colon’ and ‘rectum and anus’, (ii) ‘lung and bronchus’ and ‘prostate’, (iii) ‘breast, female’ and ‘prostate’. Although ‘colon’ and ‘rectum and anus’ should be expected to emerge as correlated, being of essentially the same tissue, the other two pairs are less expected. Breast and prostate cancer both metastasise with high affinity to the bone (Yoneda, 1998), and are both slower-progressing cancers (Peer *et al*, 1993; Barry, 2001), which may explain why the two also emerged as a highly correlated pair in terms of the metastasis network link dynamics. The correlation between lung cancer and prostate metastasis dynamics is more puzzling. However, as this analysis is looking at the links, and not the nodes, more subtle mechanisms are at play, and so perhaps more in-depth experimental research on lung and prostate cancer metastasis dynamics seems warranted.

### Topographical clustering

**Figure 3:**
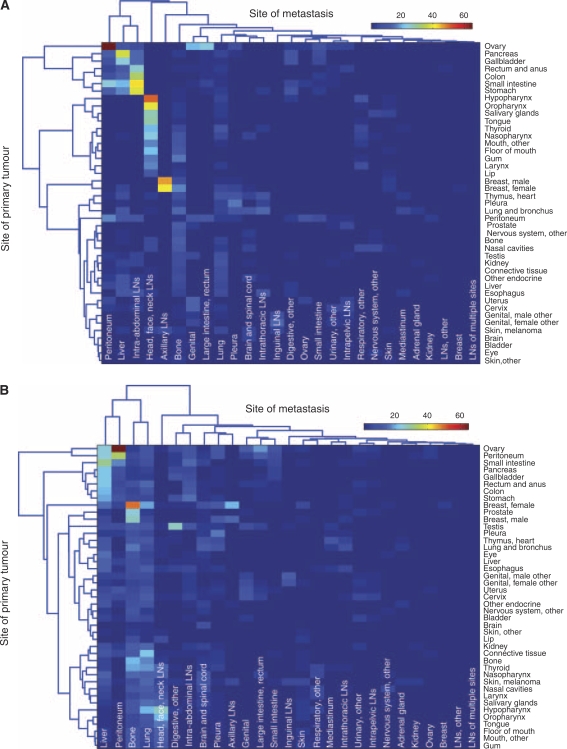
Clustergram of primary sites by characteristic sites of metastasis. (**A**), at *t*=0, the emergence of anatomical locality from this clustering is quite striking. (**B**), at *t*=48 months, a greater percentage of 2 cancers have progressed to more advanced stages, and thus the clustering is slightly different. Note: Larger high-resolution versions of these clustergrams can be found in the supplemental materials (Supplementary Figures S4 and S5).

Results of the hierarchical clustering of the sites of primary tumour and the sites of metastasis by their incidence hazard function are shown in Figure 3 (*t*=0 in Figure 3A and *t*=48 months in Figure 3B). At *t*=0, primary cancer types are clustered in three large groups with distinct patterns of metastasis development. The first group, which includes the ovary, pancreas, gallbladder, rectum and anus, colon, small intestine, and stomach, very strongly metastasise to the peritoneum, liver, and intra-abdominal lymph nodes. The second group, which includes the hypopharynx, oropharynx, tongue, thyroid, nasopharynx, floor of the mouth, gum, larynx, and lip, metastasise strongly to the lymph nodes in the head, face, and neck, and to a lesser degree, the bone and lung. The third group, which includes cancers, such as lung, prostate, bone, testis, kidney, liver, oesophagus, uterus, cervix, skin (melanoma), and others, include cancers which at *t*=0 tend to exhibit metastasis profiles with broader specificity and comparatively lower magnitudes. Breast cancer, however, is clustered by itself because of the strong affinity for the axillary lymph nodes. Through all of this, it must also be kept in mind that different cancers have differing proportions of the stages at which they are presented at diagnosis, because of the varying natural histories and different abilities in screening and detection (Halpern *et al*, 2008; Jemal *et al*, 2008). However, this clustergram reveals a distinct pattern arranged strictly by anatomical location. By *t*=48, the pattern becomes more perturbed, but much of the anatomical arrangement present earlier is still preserved.

### Prediction of the primary cancer site from a sequence of metastases

The multinomial logistic regression model achieved an overall accuracy of 51%, with most patients being classified as one of the six major cancer types. Prostate was correctly classified (true positive rate) 84% of the time, colon 80%, lung and bronchus 69%, ovary 64%, larynx 61%, and female breast 56% (Supplementary Table S3). The other cancer types had a true positive rate of <10%. Hess *et al*, 2006 developed a similar MLR algorithm for predicting the primary cancer site (nine sites) given a set of metastases. An overall accuracy of 64% was achieved, which is slightly better than the overall accuracy of our algorithm, but it must be kept in mind that we used 43 primary cancer sites (which includes many less common sites).

**Figure 4:**
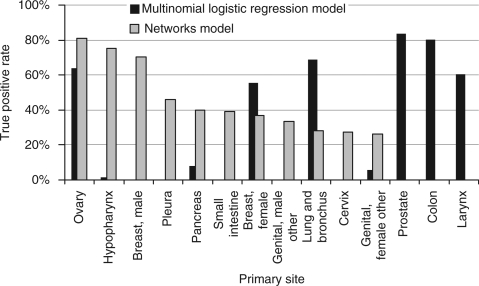
Prediction of the primary cancer site from a sequence of metastases. The primary cancer types for which the true positive rates exceed 25% from each model are shown. The multinomial logistic regression (MLR) algorithm takes into account the number of patients in the respective categories, and therefore, a relatively rare cancer type will be classified as a common cancer type with similar metastasis patterns. The MLR algorithm and the network algorithm perform in different ways: the MLR classifies everything into a few common cancer types, whereas the network algorithm is able to differentiate between rarer cancer types.

Rather than classifying patients into one of the six major cancer types, the network model for predicting the primary cancer site classifies patients into many more categories. Eleven cancer types achieved a true positive rate of >25%, most of which are less common cancers (Supplementary Table S4). For example, although almost all patients with colon cancer were classified into other categories, ovary had a true positive rate of 81%, hypopharynx 75%, male breast 70%, pleura 46%, pancreas 40%, small intestine 39%, female breast 37%, male genital 33%, lung and bronchus 28%, cervix 27%, and female genital 26%. Even though the overall accuracy may be less than that of the MLR algorithm, the network model has the advantage of broader specificity and sensitivity toward cancers of less common sites (Supplementary Table S5). The true positive rates for those sites exhibiting true positive rates >25% with either method are shown in Figure 4.

### Prediction of additional secondary cancer sites (metastases)

**Figure 5:**
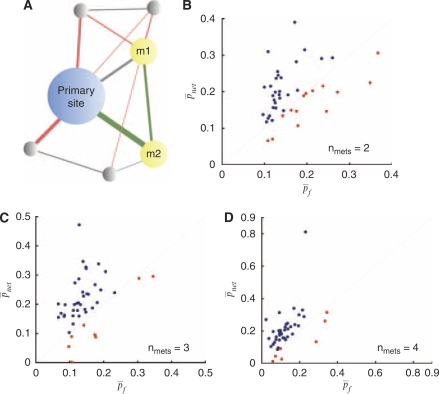
Using the cancer metastasis networks to predict the temporal sequence of additional metastases. (**A**), diagram of how *p¯*_net_ is calculated, for the case of *n*_mets_=2. To calculate the probability of developing a metastasis at site m2, given the primary cancer type represented in blue and a metastasis already having developed at site m1, the strength of the green links (represented by their widths) are summed, and divided by the summation of the strength of the red and green links. The grey links are ignored. (**B**), *p¯*_*f*_ *vs p¯*_net_, for *n*_mets_=2. (**C**), *p¯*_*f*_ *vs p¯*_net_, for *n*_mets_=3. (**D**), *p¯*_*f*_ *vs p¯*_net_, for *n*_mets_=4. Each point represents a primary cancer type. Red represents the primary cancer types for which *p¯*_net_<*p¯*_*f*_, and blue represents those for which *p¯*_net_>*p¯*_*f*_.

Although the previous method should prove helpful in the case of an occult primary neoplasm that – other than the symptomatic metastases – does not yet show on imaging, perhaps the more clinically useful prediction is the forward prediction of additional possible metastases. We therefore compared a cancer metastasis network-based algorithm and a traditional fractional method on patients with 1, 2, 3, and 4 metastases. Those results are summarised in Table 1 and Supplementary Table S6. Figure 5A is a graphical summary of the algorithm methodology. For patients with 1 metastasis, predicting *m*_1_ turns out to be no better than using the fractional method. This is expected, as the strength of those links directly connected to the primary cancer node is proportional to their respective metastasis incidences. However, with *n*_mets_>1, the algorithm with the network model performs better than the fractional method for the majority of primary cancer sites (Figures 5B–D). For *n*_mets_=2, there are 29 out of 43 primary cancer sites where *p¯*_net_/*p¯*_*f*_>1, with the average value among those being 1.525 (max: 2.858). For *n*_mets_=3, there are 35 primary cancer sites where *p¯*_net_/*p¯*_*f*_>1, with the average value among those being 1.819 (max: 3.683). For *n*_mets_=4, there are 36 primary cancer sites where *p¯*_net_/*p¯*_*f*_>1, with the average value among those being 2.119 (max: 11.619). This shows that the network captures temporal information and subtle relationships that would otherwise not be considered, and hence, allows for better-performing predictive algorithms.

## Discussion

Through a large data set of cancer patients, we have investigated the topographical patterns of clinical metastasis development using a network approach. Although the ‘seed and soil’ hypothesis (Fidler, 2003) certainly still holds, both anatomical proximity and anatomical connection seem to be dominant factors when the analysis of metastatic sites includes many more sites and many more primary cancer types. To our knowledge, such a comprehensive study has not previously been conducted, especially not one including the rarer cancer types.

Our study has shown that treating secondary metastases as separate, comorbid diseases allows the construction of cancer type-specific metastatic progression networks. From these networks, we are able to analyse the dynamics of each cancer-specific network and compare one network to another. Furthermore, we are able to use these networks as the basis of predictive algorithms, which we have shown in many cases to be better performing than conventional algorithms. We note that there are also other types of models one can build for comparison, such as a Markov model or a Cox model with time-dependent covariates. However, these may be better suited to smaller-scale studies with more detailed information on the underlying variables.

In Figure 1, we showed that the profile of hazard functions for certain types of cancer can be highly specific, such as in prostate cancer, or it can have a much broader profile, such as in bladder cancer. Broader profiles create for three possibilities. The first possibility is that these cancer types truly do have lesser selectivity in the sites of secondary dissemination. The second possibility is that the cancer type categories as defined here encompass a broad range of sub-classifications, each of which may exhibit distinct patterns by themselves. A third possibility is that these cancer types have tumours, which display more cellular heterogeneity, with different clonal populations within the tumours possessing different affinities to distant sites (Fidler, 1978, 2003).

In recent years, we have come to discover a number of molecules that drive organ specificity, but it still does not necessarily answer the question of why different types and subtypes of cancer metastasise to specific secondary sites, and with varied propensities. Consideration of the original predisposition of the transformed cell of origin suggests several possibilities that may explain these phenomena. Certain cell lineages may express molecules that bias the metastatic efficiency to various target organs. For example, both normal and cancerous mammary epithelial cells express Receptor Activator of Nuclear Factor *κ*B (RANK) – the receptor for the osteoclast differentiation factor Receptor Activator of Nuclear Factor *κ*B ligand (RANKL) (Dougall and Chaisson, 2006). Studies suggest that this receptor–ligand combination may predispose breast cancer cells to colonise bone (Roodman, 2004; Yoneda and Hiraga, 2005; Jones *et al*, 2006). The developmental history of a cell may also predispose it to activate expression of specific metastasis-promoting mechanisms on malignant transformation. Lineage-specific signalling circuits may create differential responses to the same oncogenic alterations, or developmentally imprinted epigenetic modifications may influence transcriptional accessibility of the transformed genome. Therefore, one would expect cells that are developmentally similar to act in a more similar manner than cells further apart in lineage. Indeed, in Figure 3B, we see that at *t*=48 months, a time when most cancers have progressed to an advanced stage, the clustering suggests that this developmental effect may have a role, in addition to blood flow characteristics. The clustergram groups primary sites into more or less three groups. The first group is composed of abdominal sites connected through the hepatic portal system. The second group is comprised of various sites in the torso, and the third group is comprised of various sites in the head and neck. Anatomical arguments seem to still dominate.

Although no similar large-scale study has been carried out comparing the metastasis development patterns by anatomical site of a large numbers of different cancer types, the data set we use does carry with it some limitations. The Medicare claims data contain information on 96% of Americans ⩾65 years of age (Hatten, 1980). This provides excellent coverage of an entire demographic, yet does not represent a cross-section of the population-at-large in terms of age. The data set also does not contain information on patients who were not hospitalised. Within the data, diseases were recorded in the ICD-9-CM classification scheme format and potential errors for using the ICD classification scheme at entry, and disease coding in general have been noted (Surján, 1999). However, Medicare claims data have been shown to be both accurate (Zhang *et al*, 1999; Hennessy *et al*, 2007) and sensitive (Cooper *et al*, 1999). Finally, the record of an event of metastasis is a function of its clinical detection. Metastases are not typically one or two new growths – they may number in hundreds or more, many of which are below thresholds of clinical detection. Micrometastatic disease is an important prognostic indicator (Pantel *et al*, 1999), but they will escape detection and thus not be recorded. This restricts both the spatial and temporal resolutions.

We have nonetheless been able to show that the cancer metastasis network captures important and useful temporal and relational information, and thus be able to serve as the basis for better predictive algorithms. Using a network approach, additional questions on metastatic dissemination can be explored – for example, how network properties and characteristics change by age, gender, race, disease stage, or treatment. In attempts to explore these and other questions, the Surveillance, Epidemiology and End Results (SEER)-Medicare linked data set may be a useful resource (http://seer.cancer.gov). The study of more specific networks may yield further insights into the metastatic cascade and the patterns of metastasis *per se*. In addition, coupling molecular information underlying cancer with these phenotypic networks may also prove useful, and possibly lead to better treatment of metastatic cancer. At the very least, these metastasis networks may be used to identify a likely sequence of metastases in a patient, and thus guide diagnostic tests and specific treatment targeting those sites.

## Figures and Tables

**Table 1:** Accuracy of the network model in predicting the temporal sequence of metastases, by site of primary cancer, for *n*_mets_=2 and *n*_mets_=3

	***n*_mets_=2**				***n*_mets_=3**			
**Primary site**	***N***	***p¯*_*f*_ (%)**	***p¯*_net_ (%)**	***p¯*_net_/*p¯*_*f*_**	***N***	***p¯*_*f*_ (%)**	***p¯*_net_ (%)**	***p¯*_net_/*p¯*_*f*_**
Lip	28	12.7	15.5	1.228	5	10.6	0.2	0.014
Tongue	125	12.5	19.9	1.585	46	6.7	15.5	2.321
Salivary glands	103	12.0	18.9	1.579	34	12.7	29.6	2.324
Gum	32	27.2	19.9	0.731	13	14.2	12.9	0.909
Floor of mouth	56	19.4	31.5	1.620	13	20.6	34.9	1.694
Mouth, other	94	17.5	28.2	1.614	26	10.6	9.1	0.862
Oropharynx	68	12 .3	17.7	1.445	19	7.5	16.9	2.257
Nasopharynx	40	12.5	17.0	1.356	18	14.8	32.6	2.204
Hypopharynx	104	10.8	31.0	2.858	27	11.6	27.9	2.411
Oesophagus	503	11.2	13.4	1.197	174	11.0	16.2	1.478
Stomach	1514	13.9	23.8	1.713	530	12.3	18.4	1.500
Small intestine	375	17.5	14.7	0.838	132	14.8	17.8	1.207
Colon	7681	17.1	39.0	2.282	2870	15.1	24.7	1.632
Rectum and anus	2734	15.5	19.1	1.232	963	13.8	24.4	1.764
Liver	185	19.2	18.9	0.983	57	15.5	23.7	1.531
Gallbladder	439	23.4	29.1	1.241	172	18.5	34.7	1.876
Pancreas	1438	23.7	21.5	0.907	410	16.7	20.0	1.197
Peritoneum	155	14.5	14.8	1.024	100	11.4	22.3	1.956
Nasal cavities	61	16.3	14.9	0.916	32	17.5	27.3	1.561
Larynx	241	11.4	21.5	1.878	79	8.5	16.9	1.992
Lung and bronchus	9653	14.3	13.4	0.937	3546	14.5	18.9	1.303
Pleura	67	19.7	19.5	0.989	23	14.1	22.9	1.624
Thymus, heart, and mediastinum	78	10.8	6.6	0.607	21	9.7	8.0	0.817
Bone	107	26.0	29.3	1.126	29	30.4	29.1	0.959
Connective tissue	183	13.5	20.1	1.481	63	9.9	11.1	1.124
Skin, melanoma	247	10.0	21.2	2.132	119	8.7	21.7	2.501
Skin, other	343	11.8	12.1	1.027	114	12.1	20.6	1.695
Breast, female	3129	17.8	20.4	1.148	1463	19.2	25.5	1.333
Breast, male	47	13.1	25.5	1.952	19	14.8	34.1	2.294
Cervix	310	12.0	15.6	1.300	144	11.1	18.2	1.638
Uterus	979	13.2	24.5	1.858	491	12.8	17.1	1.343
Ovary	2138	21.3	20.3	0.950	1355	18.1	22.1	1.221
Genital, female other	198	9.7	13.7	1.421	78	8.4	16.0	1.911
Prostate	5315	34.8	22.4	0.644	1321	34.5	29.4	0.851
Testis	10	13.4	19.2	1.430	6	12.8	47.2	3.683
Genital, male other	34	10.9	12.7	1.168	6	6.7	20.1	3.017
Bladder	1329	10.5	11.7	1.119	531	11.0	13.6	1.232
Kidney	1220	13.6	18.2	1.344	488	13.2	21.1	1.599
Eye	36	17.9	10.7	0.597	7	17.7	8.9	0.504
Brain	77	36.7	30.6	0.834	25	23.2	24.7	1.062
Nervous system, other	26	24.6	14.6	0.596	10	17.4	10.8	0.618
Thyroid	257	12.7	23.7	1.857	65	12.6	21.2	1.678
Other endocrine	32	11.9	7.0	0.589	9	13.9	34.7	2.485

*p¯*_*f*_ is the mean accuracy for the fractional method, *p¯*_net_ is the mean accuracy for the network model, and *p¯*_net_/*p¯*_*f*_ is their ratio. *N* is the number of patients in each case.
