# Supplementary material for: Cancer metastasis networks and the prediction of progression patterns
Source: Br J Cancer. 2009 Aug 25;101(5):749–58. doi: 10.1038/sj.bjc.6605214 (PMC2736851; doi:10.1038/sj.bjc.6605214)
Supplement: Supplementary Information [file 6605214x1.pdf]

Supplemental Information

Additional Methods

Statistical significance. For every φ-correlation, we tested the hypothesis that $\varphi \neq 0$ by a t-test, where t is calculated by:

$$t = \frac{\varphi\sqrt{\max(m_i, m_j) - 2}}{\sqrt{1-\varphi^2}} \qquad \textbf{[S1]}$$

The max function gives the most conservative value for t when the incidence of the two variables being correlated is unequal. The p-value was then calculated using Student's t cumulative distribution function with degrees of freedom $\max(m_i, m_j) - 2$. The p-value threshold for significance was set at 0.01. For every relative risk value, we estimated the significance at the 0.01 level by calculating its 99% upper and lower confidence bounds (25):

$$RR_{X,ij} \exp\left(\pm 2.56 \left(\frac{1}{C_{ij}} + \frac{1}{m_i m_j} - \frac{1}{N_X} - \frac{1}{{N_X}^2} \right) \right) \qquad \textbf{[S2]}$$

Clustering. We hierarchically clustered primary tumor types and metastasis sites using MATLAB 7.5 (MathWorks Inc., Natick, MA). Ward's linkage was used, which employs an incremental within-cluster sum of squares, defined as the sum of the squares of all distances between objects within a cluster and the cluster centroid.

Table S1. Primary cancer site and corresponding ICD-9 codes.

	Site	ICD-9 code
1	Lip	140
2	Tongue	141
3	Salivary glands	142
4	Gum	143
5	Floor of mouth	144
6	Mouth, other	145
7	Oropharynx	146
8	Nasopharynx	147
9	Hypopharynx	148
10	Esophagus	150
11	Stomach	151
12	Small intestine	152
13	Colon	153
14	Rectum and anus	154
15	Liver	155
16	Gallbladder	156
17	Pancreas	157
18	Peritoneum	158
19	Nasal cavities	160
20	Larynx	161
21	Lung and bronchus	162
22	Pleura	163
23	Thymus, heart, or peritoneum	164
24	Bone	170
25	Connective tissue	171
26	Skin, melanoma	172
27	Skin, other	173
28	Breast, female	174
29	Breast, male	175
30	Cervix	180
31	Uterus	182
32	Ovary	183
33	Genital, female other	184
34	Prostate	185
35	Testis	186
36	Genital, male other	187
37	Bladder	188
38	Kidney	189
39	Eye	190
40	Brain	191
41	Nervous system, other	192
42	Thyroid	193
43	Other endocrine	194

Table S2. Metastasis site and corresponding ICD-9 code.

	**Site**	**ICD-9 code**
1	LNs of head, face, and neck	196.0
2	Intrathoracic LNs	196.1
3	Intra-abdominal LNs	196.2
4	Axillary LNs	196.3
5	Inguinal LNs	196.5
6	Intrapelvic LNs	196.6
7	LNs of multiple sites	196.8
8	LNs, other	196.9
9	Lung	197.0
10	Mediastinum	197.1
11	Pleura	197.2
12	Respiratory, other	197.3
13	Small intestine	197.4
14	Large intestine and rectum	197.5
15	Peritoneum	197.6
16	Liver	197.7
17	Digestive, other	197.8
18	Kidney	198.0
19	Urinary, other	198.1
20	Skin	198.2
21	Brain and spinal cord	198.3
22	Nervous system, other	198.4
23	Bone	198.5
24	Ovary	198.6
25	Adrenal gland	198.7
26	Breast	198.81
27	Genital	198.82

Table S3. Confusion matrix of the multinomial logistic regression model predicting the primary cancer site from a sequence of metastases. Shown are the primary sites which yielded a true positive rate of >25%.

		Predicted site					
		Colon	Larynx	Lung and bronchus	Breast, female	Ovary	Prostate
**True site**	Colon	80.4%	0.1%	2.7%	0.6%	8.5%	4.6%
	Larynx	10.7%	60.6%	13.2%	1.6%	0.7%	6.3%
	Lung and bronchus	11.9%	1.6%	68.9%	1.5%	0.6%	14.3%
	Breast, female	8.9%	0.4%	14.6%	55.7%	1.6%	17.8%
	Ovary	20.8%	0.1%	3.3%	0.7%	63.8%	6.1%
	Prostate	6.3%	0.3%	6.5%	0.4%	1.3%	83.7%

Table S4. Confusion matrix of the networks model predicting the primary cancer site from a sequence of metastases. Shown are the primary sites which yielded a true positive rate of >25%.

		Predicted Site										
		Hypo-pharynx	Small intestine	Pancreas	Lung and bronchus	Pleura	Breast, female	Breast, male	Cervix	Ovary	Genital, female other	Genital, male other
**True Site**	Hypo-pharynx	75.2%	0.6%	0.8%	1.1%	0.3%	3.8%	0.1%	0.0%	0.3%	0.0%	0.1%
	Small intestine	0.4%	39.0%	10.2%	0.4%	0.3%	1.5%	0.1%	1.2%	34.4%	0.2%	0.0%
	Pancreas	0.7%	15.5%	39.8%	0.7%	1.1%	1.8%	0.1%	0.1%	23.5%	0.1%	0.1%
	Lung and bronchus	3.6%	1.7%	11.3%	28.0%	13.8%	15.5%	0.6%	0.1%	1.4%	0.0%	0.1%
	Pleura	0.7%	1.0%	3.1%	8.6%	46.1%	10.8%	0.1%	0.0%	5.8%	0.0%	0.0%
	Breast, female	2.2%	1.2%	2.2%	1.5%	2.9%	36.8%	29.4%	0.3%	3.0%	0.1%	0.2%
	Breast, male	0.7%	0.7%	0.7%	1.3%	1.6%	15.8%	70.3%	0.0%	1.2%	0.1%	0.0%
	Cervix	2.1%	4.3%	2.9%	0.8%	0.7%	5.6%	0.6%	27.2%	37.0%	1.0%	1.1%
	Ovary	0.3%	3.0%	3.6%	0.4%	2.6%	1.0%	0.2%	1.6%	81.1%	0.3%	0.1%
	Genital, female other	1.1%	2.1%	2.3%	0.3%	0.8%	5.4%	0.6%	13.4%	19.2%	26.4%	14.3%
	Genital, male other	1.9%	2.1%	2.4%	0.5%	0.8%	6.4%	0.8%	16.4%	8.5%	11.9%	33.4%

Table S5. Comparisons of the diagonals (true positives) of the confusion matrices of the multinomial logistic regression (MLR) model and the networks model predicting the site of primary cancer given a sequence of metastases.

Primary site	MLR model	Networks model
Lip	0.000%	1.304%
Tongue	2.793%	3.783%
Salivary glands	5.190%	4.574%
Gum	0.000%	5.333%
Floor of mouth	0.000%	1.124%
Mouth, other	0.000%	0.380%
Oropharynx	0.000%	3.279%
Nasopharynx	0.000%	7.349%
Hypopharynx	1.676%	75.188%
Esophagus	0.153%	1.238%
Stomach	4.885%	7.850%
Small intestine	0.000%	39.017%
Colon	80.368%	0.000%
Rectum and anus	1.757%	1.638%
Liver	0.000%	0.000%
Gallbladder	0.000%	12.007%
Pancreas	8.081%	39.790%
Peritoneum	0.000%	9.884%
Nasal cavities	6.431%	14.975%
Larynx	60.646%	0.096%
Lung and bronchus	68.930%	27.974%
Pleura	0.000%	46.143%
Thymus, heart, or peritoneum	0.000%	15.450%
Bone	0.000%	19.247%
Connective tissue	0.000%	13.003%
Skin, melanoma	2.860%	1.912%
Skin, other	1.030%	0.000%
Breast, female	55.695%	36.843%
Breast, male	0.000%	70.254%
Cervix	0.000%	27.237%
Uterus	6.038%	0.423%
Ovary	63.836%	81.083%
Genital, female other	5.809%	26.407%
Prostate	83.687%	0.006%
Testis	0.000%	19.540%
Genital, male other	0.000%	33.422%
Bladder	2.796%	1.203%
Kidney	5.222%	7.533%
Eye	0.000%	10.616%
Brain	0.000%	14.320%
Nervous system, other	0.000%	1.190%
Thyroid	0.691%	0.933%
Other endocrine	0.000%	5.338%

Table S6. Accuracy of networks model in predicting the temporal sequence of metastases, by primary cancer site, for n_{mets} = 1 and n_{mets} = 4. $\overline{p}_f$ is the mean accuracy for the fractional method, $\overline{p}_{net}$ is the mean accuracy for the networks model, and $\overline{p}_{net}/\overline{p}_f$ is their ratio. *N* is the number of patients in each case.

**Primary site**	**n_{mets} = 1**				**n_{mets} = 4**			
	N	$\overline{p}_f$	$\overline{p}_{net}$	$\overline{p}_{net}/\overline{p}_f$	*N*	$\overline{p}_f$	$\overline{p}_{net}$	$\overline{p}_{net}/\overline{p}_f$
Lip	92	31.5%	14.0%	0.445	1	17.5%	23.9%	1.364
Tongue	750	49.5%	29.1%	0.589	11	6.1%	11.8%	1.924
Salivary glands	395	30.7%	9.0%	0.294	11	9.4%	31.4%	3.354
Gum	168	32.4%	17.4%	0.536	2	2.3%	27.1%	11.619
Floor of mouth	323	35.7%	20.2%	0.565	4	3.8%	15.3%	3.985
Mouth, other	389	31.5%	19.0%	0.605	6	7.4%	4.4%	0.595
Oropharynx	348	50.8%	26.8%	0.528	5	16.3%	18.1%	1.113
Nasopharynx	120	23.2%	20.2%	0.869	3	19.5%	23.2%	1.194
Hypopharynx	401	41.4%	17.0%	0.411	9	6.1%	19.7%	3.210
Esophagus	1201	11.4%	10.3%	0.900	52	12.2%	20.7%	1.690
Stomach	3739	22.2%	51.1%	2.303	174	9.5%	17.5%	1.837
Small intestine	793	21.9%	5.9%	0.271	51	10.0%	19.3%	1.942
Colon	23370	26.5%	5.5%	0.207	981	12.5%	22.5%	1.802
Rectum and anus	8386	19.3%	6.5%	0.335	352	9.9%	20.4%	2.059
Liver	745	21.1%	26.0%	1.229	15	10.0%	9.1%	0.914
Gallbladder	1139	24.5%	13.2%	0.540	49	17.0%	34.1%	1.998
Pancreas	4036	26.9%	11.2%	0.415	128	13.7%	16.4%	1.197
Peritoneum	328	14.0%	15.4%	1.101	46	13.0%	24.2%	1.864
Nasal cavities	197	13.8%	14.6%	1.058	7	21.6%	33.0%	1.532
Larynx	1166	27.4%	20.6%	0.752	24	7.7%	16.4%	2.119
Lung and bronchus	24905	13.1%	7.0%	0.533	1090	13.9%	21.3%	1.533
Pleura	261	26.1%	17.0%	0.651	5	12.7%	14.6%	1.154
Thymus, heart, and mediastinum	178	11.3%	3.4%	0.300	12	8.4%	8.6%	1.027
Bone	335	25.6%	40.4%	1.574	8	28.7%	13.4%	0.466
Connective tissue	493	16.3%	25.5%	1.566	15	10.5%	18.2%	1.735
Skin, melanoma	691	9.3%	6.8%	0.732	52	7.6%	14.7%	1.924
Skin, other	1293	10.7%	9.0%	0.839	22	9.4%	14.5%	1.538
Breast, female	7081	21.2%	17.3%	0.818	595	19.7%	30.0%	1.524
Breast, male	233	45.4%	6.4%	0.140	3	9.5%	18.6%	1.956
Cervix	894	14.2%	16.4%	1.156	46	8.7%	9.6%	1.095
Uterus	3320	17.1%	6.1%	0.356	236	12.0%	15.6%	1.306
Ovary	3194	28.3%	7.7%	0.273	703	15.4%	22.7%	1.474
Genital, female other	577	17.9%	12.6%	0.706	21	6.8%	12.9%	1.895
Prostate	26642	34.5%	16.8%	0.486	347	34.4%	31.5%	0.917
Testis	28	10.3%	11.6%	1.131	2	23.1%	81.1%	3.505
Genital, male other	130	22.2%	43.8%	1.976	5	5.9%	1.2%	0.204
Bladder	4267	10.8%	9.5%	0.877	174	11.3%	15.8%	1.404
Kidney	2891	12.3%	8.7%	0.709	175	13.7%	24.8%	1.810
Eye	101	19.7%	7.4%	0.374	4	22.4%	28.9%	1.295
Brain	301	45.7%	25.1%	0.550	8	33.4%	26.2%	0.783
Nervous system, other	86	24.3%	2.8%	0.117	2	5.0%	10.4%	2.091
Thyroid	777	23.0%	12.9%	0.561	29	9.1%	20.2%	2.223
Other endocrine	98	12.1%	5.8%	0.474	8	10.6%	2.6%	0.242

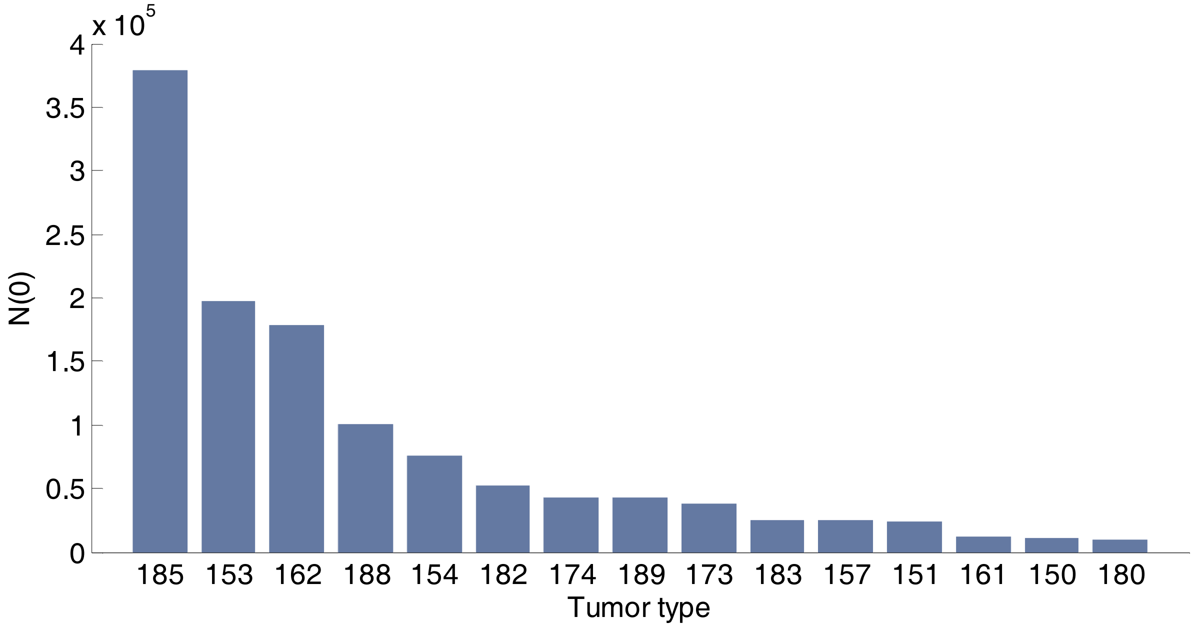

Figure S1. Number of diagnoses at time t=0, by primary site or tumor type (represented by the three-digit ICD-9 code). Note: t is defined as the time from the first diagnosis of a primary cancer. The primary sites that have the largest number of diagnoses within the dataset are (in descending order): prostate, colon, lung, and bladder.

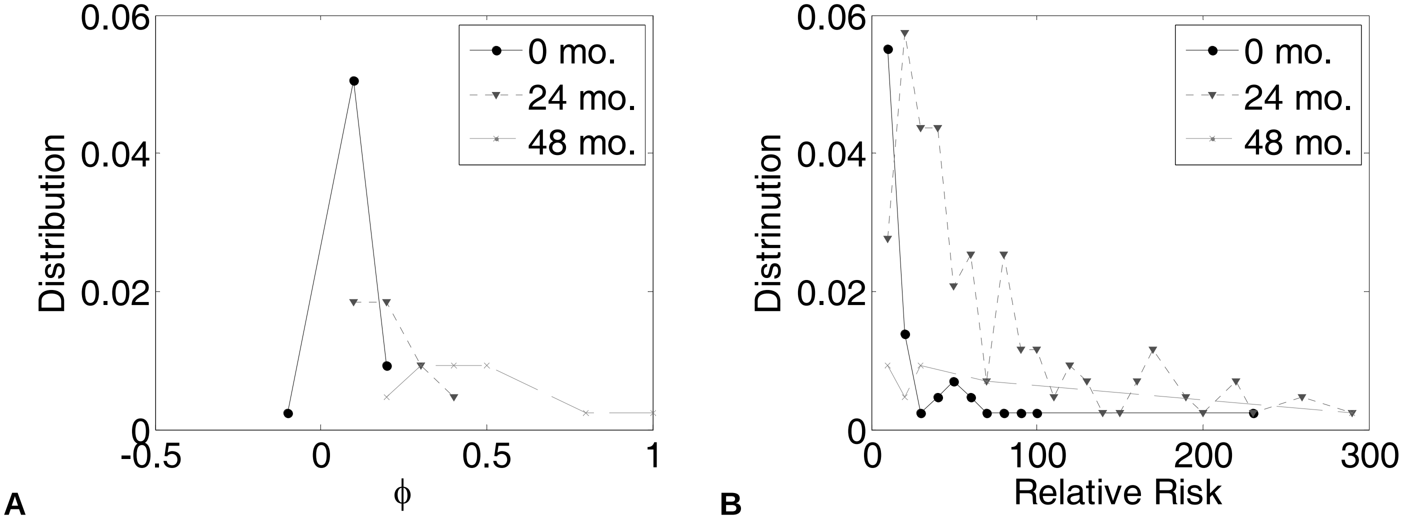

Figure S2. Distributions of *A*, phi and *B*, relative risk values over time, for colon cancer. Colon cancer was chosen as a representative example. As time progresses, the peaks of the phi distribution shift toward higher values of phi and the distributions encompass a much larger range. It is less obvious how the relative risk distribution behaves over time, but it appears that the RR distribution also shifts toward higher values.

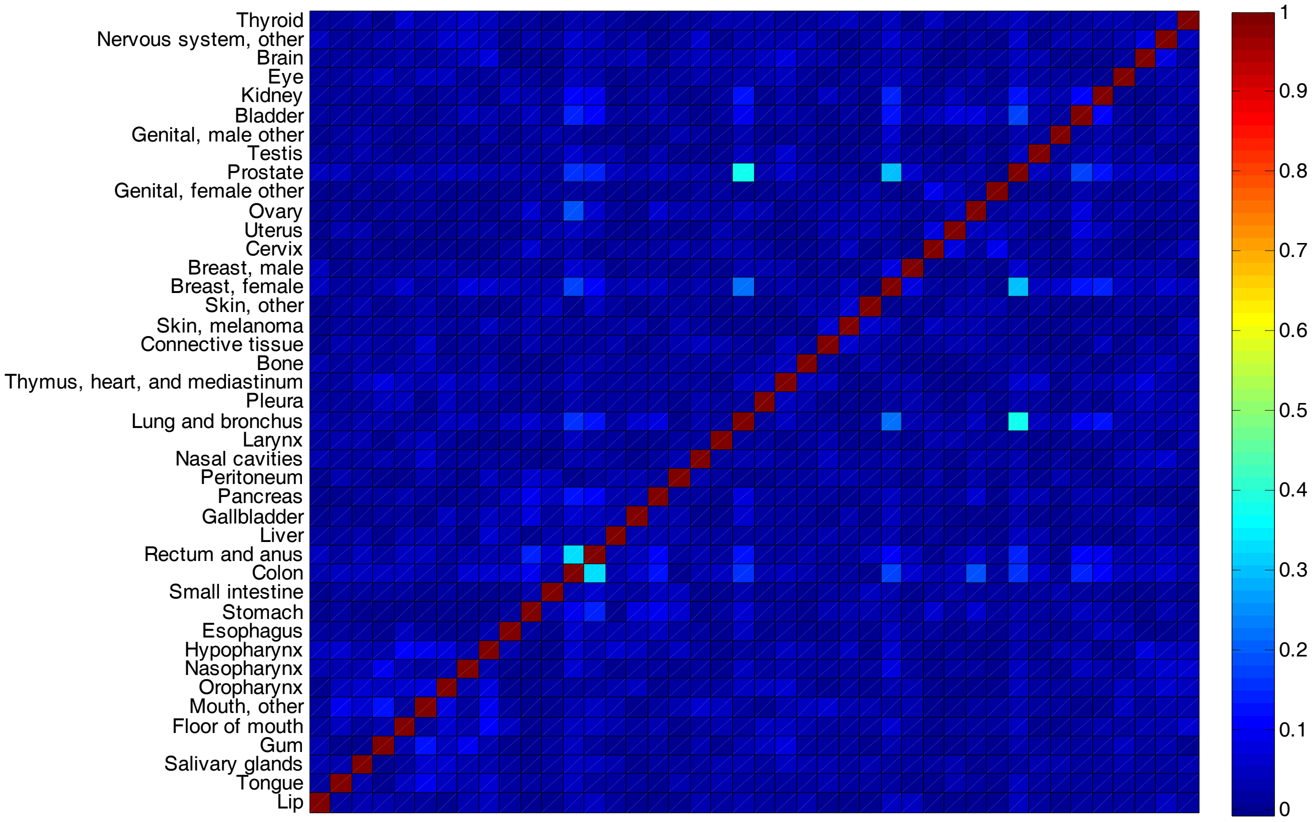

Figure S3. Correlation coefficient matrix by primary tumor type based on metastasis link dynamics. Using the phi measure and with significance set at 0.01, we measured the pair-wise correlations between metastasis network links for every primary cancer type. While the vast majority of primary cancer types exhibit low correlation values with one another based on this, a few do stand out: (i) ‘colon’ and ‘rectum and anus’, (ii) ‘lung and bronchus’ and ‘prostate’, (iii) ‘breast, female’ and ‘prostate’.

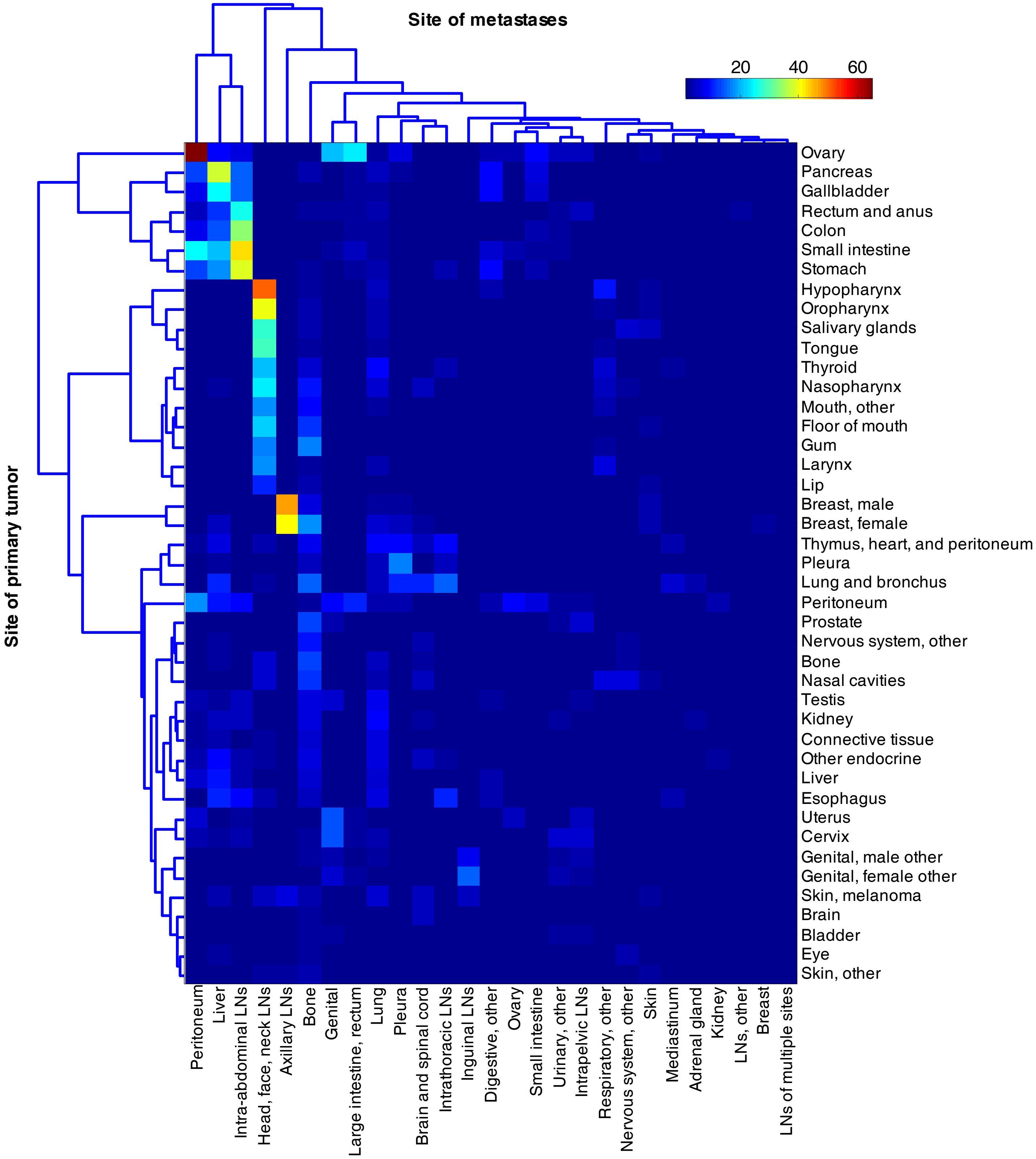

Figure S4. Clustergram of primary tumor types by characteristic sites of metastasis, at t=0.

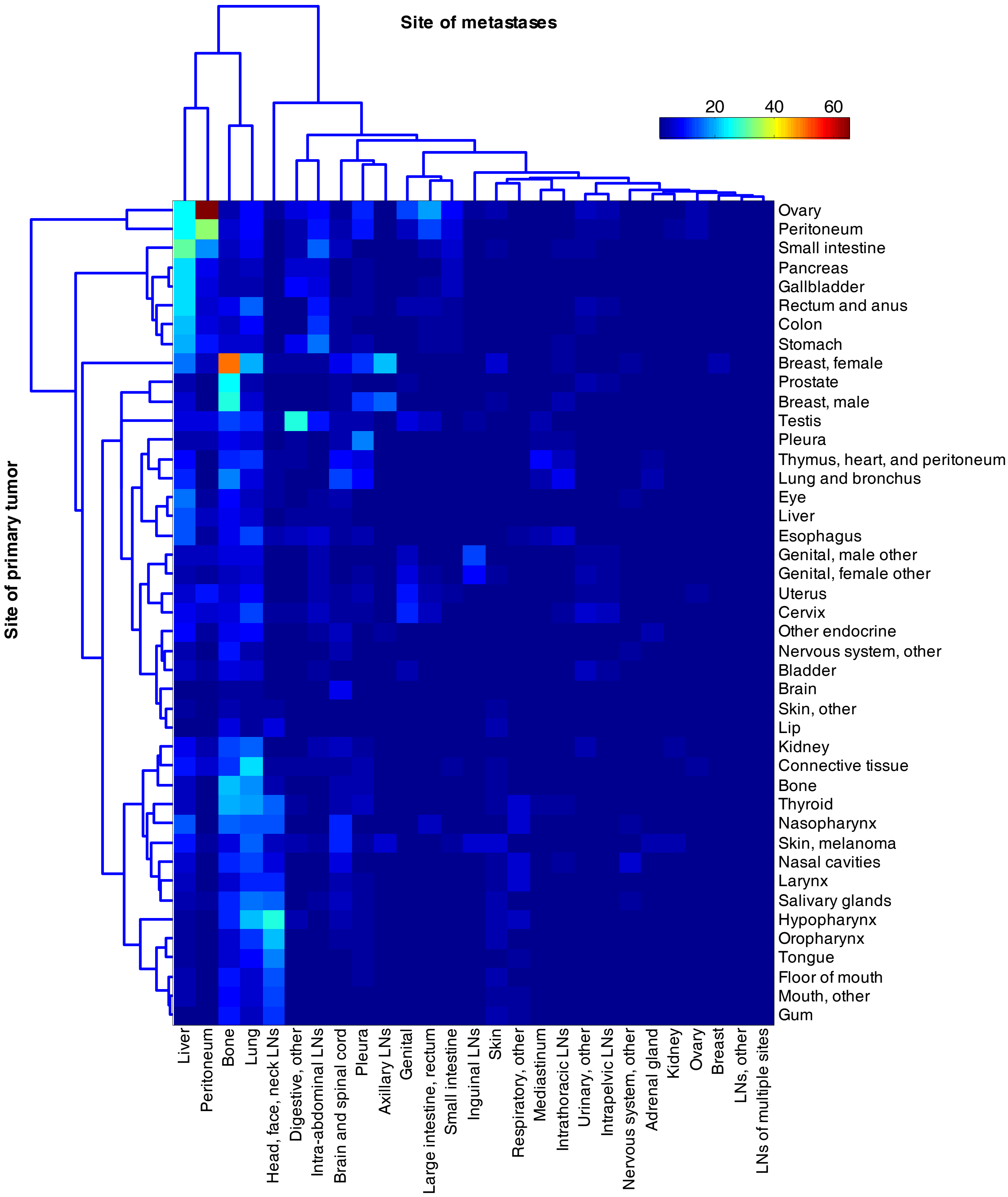

Figure S5. Clustergram of primary tumor types by characteristic sites of metastasis, at t=48 months.
